# Influence of the Degree of Fruitiness on the Quality Assessment of Virgin Olive Oils Using Electronic Nose Technology

**DOI:** 10.3390/s24082565

**Published:** 2024-04-17

**Authors:** Javiera P. Navarro Soto, Sergio Illana Rico, Diego M. Martínez Gila, Silvia Satorres Martínez

**Affiliations:** 1 System Engineering and Automation Department, University of Jaén, 23071 Jaén, Spain; jpns0002@red.ujaen.es (J.P.N.S.); sillana@ujaen.es (S.I.R.); satorres@ujaen.es (S.S.M.); 2 University Institute of Research on Olive Groves and Olive Oils, University of Jaén, 23071 Jaén, Spain

**Keywords:** e-nose, fruitiness, quality assessment, EVOO, VOO

## Abstract

The electronic nose is a non-invasive technology suitable for the analysis of edible oils. One of the practical applications in the olive oil industry is the classification of virgin oils based on their sensory characteristics. Notwithstanding that this technology, at this stage, cannot realistically replace the currently used methods, it is fruitful for a preliminary analysis of the oil quality. This work makes use of this technology to develop a methodology for the detection of the threshold by which an extra-virgin olive oil (EVOO) drops into the virgin olive oil (VOO) category. With this aim, two features were studied: the level of fruitiness level and the type of defect. The results showed a greater influence of the level of fruitiness than the type of defect in the determination of the detection threshold. Furthermore, three of the sensors (S2, S7 and S9) of the commercial e-nose PEN3 were identified as the most discriminating in the classification between EVOO and VOO oils.

## 1. Introduction

Virgin olive oil (VOO) consumption has greatly increased over the past few years and this trend is expected to continue. One of the drivers behind this change is the proven scientific evidence about its benefits for human health [1]. Boosted by the consumers’ demand for a better quality product, its production is being marked by the growing importance of extra-virgin olive oil (EVOO), an oil of supreme quality [2]. It has also increased the demand for virgin olive oil (VOO), a second category of olive oil that has a moderate presence of defects [3]. Both categories have nutritional properties that are lacking in other refined oils such as olive oil (OO), or seed oils (sunflower, canola, rapeseed, palm) [4].

This has led to a greater interest in understanding the sensory quality of virgin oil, its positive attributes and the potential faults in the whole chain of the productive process, since these could reduce the product quality [5]. In addition, it should be noted that throughout the campaign, the condition of the fruit varies, and this results in the production of oils with different qualities: EVOO, VOO and lampante (LOO). In the case of the third quality category, the LOO, this cannot be consumed directly due to the huge presence of defects, which are related to the deplorable state of the olive fruit or to the mishandling of the fruit or oils [6].

Mixing between EVOO and VOO is a common practice to increase company profitability or to achieve an olive oil with a special flavour. The labelling of these mixtures will depend on the detection or not of a defect in the oil by the official panel.

Currently, the quality of olive oil is evaluated in accordance with European legislation, in which classes or categories of oils are established by chemical analyses accompanied by organoleptic sensory analyses [7]. Sensory analyses of olive oils have to be carried out by both smell and taste assessments by means of a panel test. It is composed of a group of 8 to 12 trained persons, who identify and measure the different positive and negative sensations perceived according to codified rules and under controlled conditions. This methodology is expensive, and their results are delayed by days or weeks due to the impossibility of tasting more than 36 samples per day, which is utterly insufficient to comply with the requirements throughout the season [8].

In view of the above, there is a need to develop accurate instrumental techniques capable of performing measurements in real-time and of generating the same information as a panel in a reproducible and stable way in order to assess the quality of the VOOs rapidly and efficiently [9,10].

An alternative analytical approach, which meets the above needs, can be performed using an electronic nose. This technology is able to produce a response from the entire volatile fraction simultaneously, generating a signal according to the volatile’s intensity [11,12,13]. It requires a previous training step, which is fundamental to the experimental design, and the use of machine learning techniques to generate the correct interpretation of the volatile fingerprint [14].

Studies have demonstrated the capability of the e-nose in olive oil quality assessment, showing good performance when separating the VOOs (EVOO and VOO) from the LOO [8,15,16,17,18]. This means that it is a technology suitable for detecting a high level of defects, above 3.5/10, in olive oils. However, the detection of a low concentration of defects in oils, below 3.5/10, is a challenge not only for an electronic nose but also for an officially trained panellist [19]. This concentration of defects corresponds to the classification between EVOO and VOO, and scientific contributions are beginning to be made in this regard [20].

Researchers claim that the type of defect could be one of the main factors affecting the correct classification between EVOO and VOO [21,22,23]. In [21], it was suggested that positive attributes present in the oils could also interfere with the classification results. The most important was the fruitiness, reminiscent of olive fruit in healthy conditions, which presents as a complex mixture of volatiles capable of displaying a wide variety of aromas [24]. In relation to this attribute, recent work with an e-nose explored the prediction of the level of fruitiness in VOOs with highly accurate results [25]. In addition, a lab-made e-nose presented a great performance for fruitiness intensity discrimination in EVOOs [26].

The foregoing indicates that the e-nose is a promising technology for olive oil quality determination, and further studies should be designed to analyse to what extent the fruitiness attribute could influence measurements by this sensor. In this context, the novelty of the work presented in this paper resides in the detection of the threshold by which a higher-category oil, EVOO, passes into a lower category, i.e., VOO, due to the identification of defects by a commercial e-nose. Also analysed is how the level of fruitiness of EVOO influences the final detection threshold when mixing. Furthermore, the study considers the influence of certain kind of defects, present in the VOOs, in the estimation of the detection threshold.

The rest of the paper is organized as follows: the next section details the materials and methods employed to obtain the results discussed in Section 3. The paper ends with the conclusions presented in Section 4.

## 2. Materials and Methods

A commercial electronic nose was used to measure various samples of EVOO and VOO, hereafter called pure samples. This sensor was also used to measure mixtures of EVOOs with different percentages of VOOs, hereafter called dilutions. The origin of the pure samples, how the dilutions were prepared and how the data were acquired and further processed are detailed below.

### 2.1. Olive Oil Pure Samples

Six olive oil samples, stored in 500 mL dark-coloured bottles, were provided by the accredited laboratory CM Europa S.L., located in the province of Jaén (Spain). Samples were required to be similar in terms of chemical characteristics. This avoided any interference in the attributes studied, the level of fruitiness and the type of defect. According to the chemical analysis of the selected oils for this study, all of them belonged to the extra-virgin class. However, the organoleptic analysis distinguished between two types of oil classes (Table 1). 

According to the organoleptic analysis, and in compliance with the European legislation [7], all of them had a level of fruitiness above zero, which is a positive attribute and must be present in all categories of VOOs. Four of the samples were EVOOs, which also had to contain zero defects. The other two remaining samples belonged to the VOO class, characterized by the presence of a defect level above 0 but below 3.5.

Among the four EVOOs, there were notable differences in the level of fruitiness. Those with a level above 5.5 were considered very fruity and labelled as H1 and H2. On the contrary, those with a level below 3.5 were considered low fruity and labelled as L1 and L2. This difference in fruitiness is one of the factors studied in this research.

In order not to interfere with the experiment, the level of fruitiness of the two VOOs was very low and similar for both. The main difference between the VOO samples was the type of defect in each of them. The one with a musty-type defect was designated D1, while the one with a fusty-type defect was designated D2. The musty defect is a characteristic flavour of oils obtained from olives that have been piled under humid conditions for several days, with the consequence of the development of various kinds of fungi [24]. The fusty defect occurs when harvested olives are stored incorrectly and for a longer period than advisable. It is the characteristic flavour of oils obtained from olives in an advanced stage of fermentation [24].

### 2.2. Olive Oil Mixtures and Dilutions

For the experimental study, a total of 192 samples were used. These were created from six matrix oils with different organoleptic characteristics to check to what extent the e-nose could recognize them as an EVOO or a VOO. Each of the four EVOOs was mixed with each of the two VOOs. The result was eight different mixtures (Table 2). 

For each of the mixtures, a set of serial dilutions was prepared in descending proportions according to Table 3, following the proposal of [23] Lerma García et al., 2010. Each of the dilutions, together with the pure samples needed to create them, was designated as a sample. Each sample was stored in a 13.5 mL glass vial, and the total amount per vial was 5 g of oil, leaving half of the vial space free for volatile compounds (VOCs). In order to keep the samples well preserved before the measurement procedure, vials were sealed with a hermetic rubber cup and stored in the dark in a 21 °C room. 

For each of the mixtures in Table 2, a set of dilutions was made according to the proportions shown in Table 3. This made a total of 96 samples (12 samples × 8 mixtures). To increase the accuracy, a complete replication of the 96 samples was created. Therefore, the final number of samples considered in this experiment was 192 (32 pure samples plus 160 dilution samples).

### 2.3. Instrumentation and Working Conditions

The analysis of the headspace of each vial was performed using an electronic nose model, PEN3 (Airsense Analytics GmbH, Schwerin, Germany), with the integrated software Win Muster v. 1.6.2. This device has a gas sampling unit with a maximum flow rate of 600 mL/min and an integrated sensor array composed of 10 different thermo-regulated (200–500 °C) metal oxide thick film sensors (MOS) sensitive to different classes of chemical compounds. Specifically, the MOSs and the sensing capability of each of them are as follows: S1 (aromatics), S2 (broad-range), S3 (aromatics and ammonia), S4 (hydrogen), S5 (alkanes or aliphatics and less-polar compounds), S6 (broad methane), S7 (sulphur and terpenes), S8 (broad alcohols and aromatics), S9 (sulphur chlorinate) and S10 (methane aliphatic) [11].

The order in which the samples were measured by the e-nose was selected randomly. To standardise the measuring conditions, vials were opened ten minutes before measurement to free all the VOCs already present. After that, at five minutes before measurement, the vials were completely sealed with a pierceable silicon/Teflon layer (in a similar procedure as that described in [15,23].

The vials were put under a warm bath conditioned to 40 °C for one minute before taking the measurement. This process increased the oil temperature up to 28–29 °C, ensuring the appropriate volatilization of all the potential VOCs. The time period was chosen after several assays, which not only improved the VOC signal but also shortened the measurement time compared with other related studies [11,21,23]. This avoided a long time of exposure to air and temperature, which are oxidative factors that could interfere with and lead to the deterioration of oil aromas. Finally, the room temperature was always kept between 22 °C and 23 °C.

Since the pure oil samples were frozen before the dilutions were made, they had almost no moisture, and only a purifying filter for external odours was need. The filter used was a vial glass of 100 mL, filled with activated carbon. This type of filter cleans the external air that flows directly into the e-nose sensor chamber. The cleaning stage lasts 60 s and was performed just before each sample measurement.

**Figure 1:**
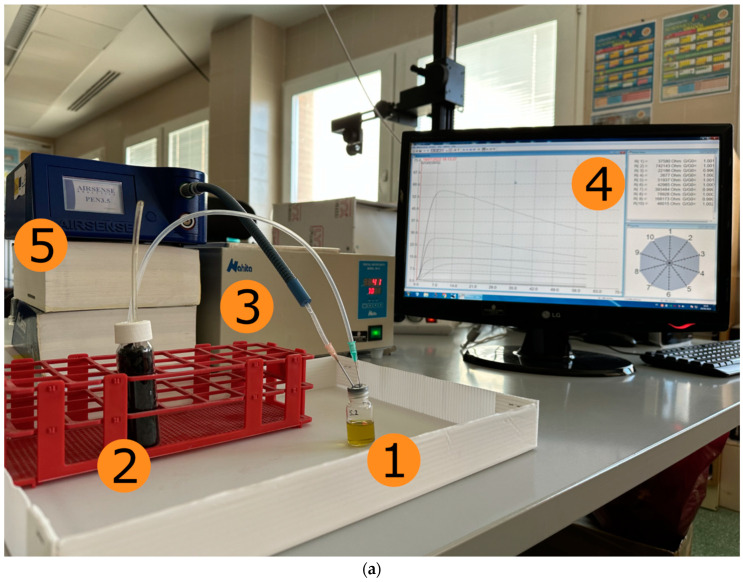

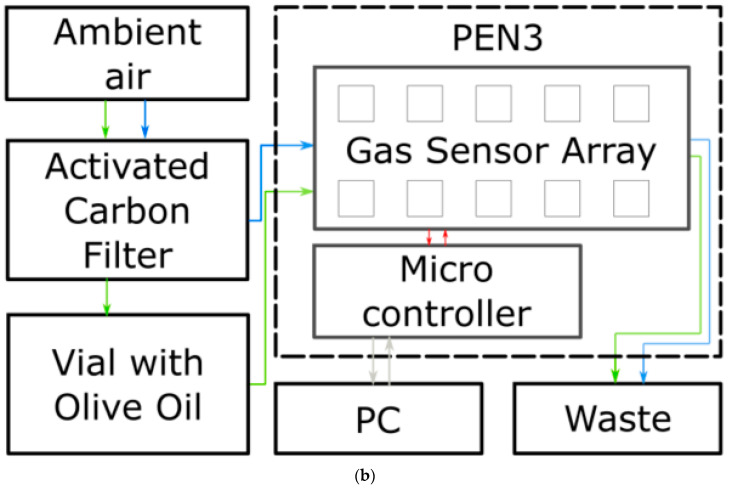
(**a**) Experimental set-up of the electronic nose used in sample measurements (1 = tested sample; 2 = activated-carbon filter; 3 = water heater; 4 = data acquisition software; 5 = PEN3 e-nose). (**b**) Structural diagram of the electronic nose system. The blue arrows indicate the air flow in the chamber cleaning phase; the green lines indicate the air flow in the measurement process; the red arrows show the electronic signals exchanged between the sensor chamber and the PEN3 microcontroller; finally, the grey arrows indicate the data exchanged between the microcontroller and the PC.

After the air passes through the activated-carbon filter, it flows through the headspace of the vial and then into the array or sensors. When the sample volatile compounds reacts with the sensing film of the sensor, an oxygen exchange occurs, resulting in a change of electrical conductivity that is detectable by a transducer element (electrode) attached to each sensor. The signal response of each sensor was set for 60 s and sampled and recorded every second. The air flow rate for the cleaning and measurement stages was 400 mL/min. Figure 1a shows the experimental set-up of the electronic nose used in the sample measurements, and Figure 1b shows its structural diagram.

### 2.4. Data Analysis

As mentioned before, the e-nose is made up of 10 MOS-type sensors. Due to the measurement process, 60 data points were available for each oil sample. These values were normalized based on the final resistance value of the cleaning phase. The acquired data set was normalized in the hypermatrix $x_{n,s,t}$ according to Equation (1).

(1) $$x_{n,s,t}=\frac{R_{n,s,t}^{MP}}{R_{n,s,60}^{CP}}$$

where $R_{n,s,t}^{MP}$ is the electrical resistance value during the measurement phase (MP) of sample n, obtained from sensor s at time t. And $R_{n,s,60}^{CP}$ is the final value of the electrical resistance acquired during the cleaning phase (CP).

Next, a total of 6 features (Equation (2)) were computed for each oil sample and sensor. These were obtained from the hypermatrix $x_{n,s,t}$ as follows: the maximum value, the sum of all the values, the minimum value, the difference between the maximum value and the minimum value, the final value and the average value.

(2) $$v_{n,s,f}=\left[\begin{array}{c} v_{n,s,1}=max\left(x_{n,s,t}\right)\\ v_{n,s,2}=\sum_{t=1}^{60}\left(x_{n,s,t}\right)\\ v_{n,s,3}=min\left(x_{n,s,t}\right)\\ v_{n,s,4}=max\left(x_{n,s,t}\right)-min\left(x_{n,s,t}\right)\\ v_{n,s,5}=x_{n,s,60}\\ v_{n,s,6}=\frac{\sum_{t=1}^{60}\left(x_{n,s,t}\right)}{60} \end{array}\right]$$

The next step was to identify the most discriminating features, that is, those that provide more information about the quality of the oil. For this, two groups were created: the first group was formed by the hypermatrix corresponding to the measurements applied to the pure samples of EVOO ($v_{n,s,f}^{EVOO}$), and a second group was made up of the hypermatrix corresponding to the measurements of the pure VOO samples ($v_{n,s,f}^{VOO}$). Considering these two groups, a one-way ANOVA test was applied. This test enables us to find out whether different groups of an independent variable have different effects on the response variable. In our case, the response variable was the quality label of the oil. Then, ANOVA compares the variation between classes to the variation within classes. If the ratio of the between-class variation to the within-class variation is significantly high, then we can conclude that the class means are significantly different from each other. This was measured using a test statistic that has an F-distribution with $\left(k-1,N-k\right)$ degrees of freedom (Equation (3)).

(3) $$F_{s,f}=\frac{\frac{SSR_{s,f}}{k-1}}{\frac{SSE_{s,f}}{N-k}}$$

where SSR (Equation (4)) is the sum of squares due to the between-classes effect (variation between classes), SSE (Equation (5)) is the sum of squared errors (variation within classes), k is the number of groups and N is the total number of observations

(4) $$SSR_{s,f}=\sum_{c=1}^{2}{\left({\bar{y}}_{.,c,s,f}-{\bar{y}}_{.,.,s,f}\right)}^{2}$$

(5) $$SSE_{s,f}=\sum_{n=1}^{32}\sum_{c=1}^{2}{\left(y_{n,c,s,f}-{\bar{y}}_{.,c,s,f}\right)}^{2}$$

where:

(6) $$y_{n,1,s,f}=v_{n,s,f}^{EVOO}$$

(7) $$y_{n,2,s,f}=v_{n,s,f}^{VOO}$$

(8) $${\bar{y}}_{.,1,s,f}=\frac{\sum_{n=1}^{16}v_{n,s,f}^{EVOO}}{16}$$

(9) $${\bar{y}}_{.,2,s,f}=\frac{\sum_{n=1}^{16}v_{n,s,f}^{VOO}}{16}$$

(10) $${\bar{y}}_{.,.,s,f}=\frac{\sum_{n=1}^{16}v_{n,s,f}^{EVOO}+\sum_{n=1}^{16}v_{n,s,f}^{VOO}}{32}$$

If the *p*-value for the F-statistic is smaller than the significance level, then the test rejects the null hypothesis that all group means are equal and concludes that at least one of the group means is different from the others. In our case, significant features were considered when the *p*-value was less than 0.01. The sensors that obtained the highest number of significant features were selected to build the classification model.

The classification model used for the other 160 dilution samples’ classification was a nominal-type decision tree. A decision tree is a flowchart-like structure in which each internal node represents a “test” on an attribute, each branch represents the outcome of the test and each leaf node represents a class label. The paths from root to leaf represent classification rules. The performance of the classifier was evaluated using a leave-one-out cross-validation based on k-fold cross-validation, with k being the size of the dataset. K-fold cross-validation is a procedure used to estimate the performance of a machine learning algorithm when making predictions on data not considered during model training. This means that k-fold cross-validation involves fitting and evaluating k models. This, in turn, provides k estimates of a model’s performance on the dataset.

**Figure 2:**
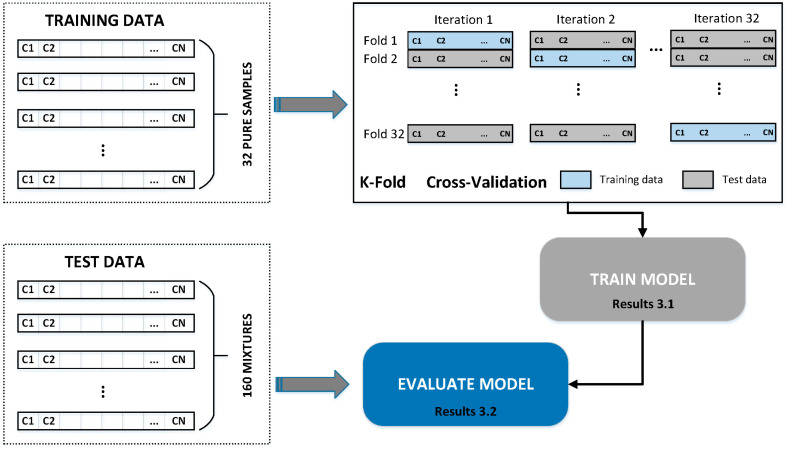
Design of the classification algorithm.

Figure 2 shows the design of the classifier, and part of the next section will describe details of the training and the evaluation of the classification model. It can be seen that the model was initially trained with the 32 pure oil samples and finally used to predict the class of the 160 mixtures.

## 3. Results and Discussion

This section presents and comments on the results obtained for the assessment of the pure samples and the olive oil dilutions.

### 3.1. Quality Assessment of Pure Samples

**Figure 3:**
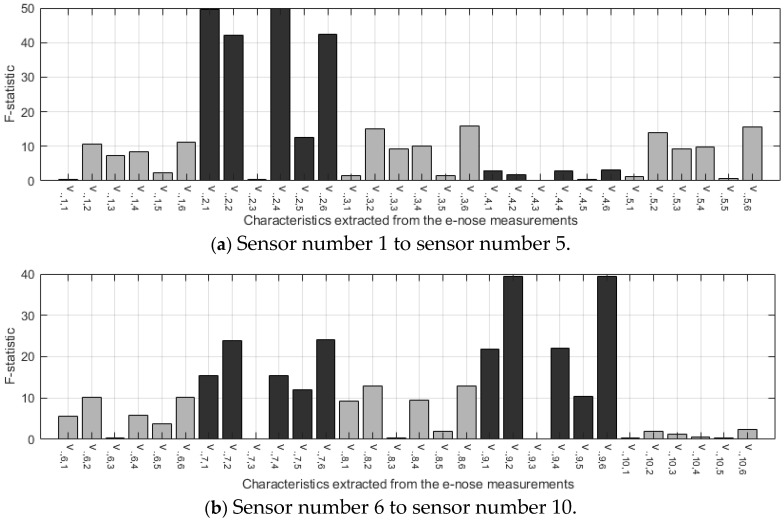
F-statistic calculated for each of the six features computed from the e-nose response of each of the ten sensors.

**Figure 4:**
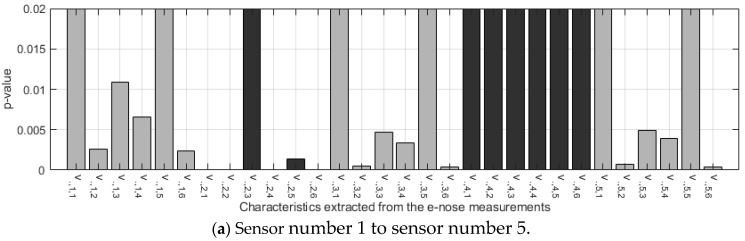

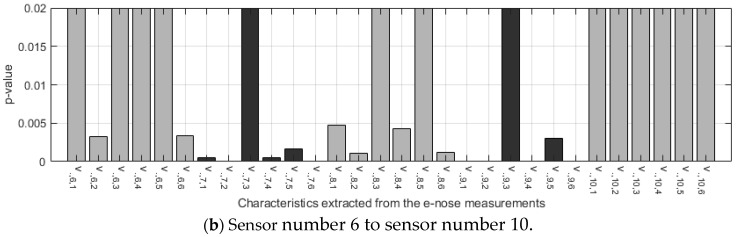
Statistical significance of the F-statistic represented by the *p*-value.

To identify the most discriminant characteristics between VOO and EVOO, the ANOVA test was applied to the characteristics extracted from the e-nose measurements performed on the pure samples. Two parameters were obtained as a result of this test: the F-statistic (Figure 3) and the statistical significance p (Figure 4).

The F statistic is related to the separability of the classes, and the higher the value, the greater the discriminatory potential. Figure 3a shows that sensor 2 is the sensor with the highest number of features with high discriminatory potential, followed by sensor 9 and sensor 7 (Figure 3b). Furthermore, the feature with the highest value is number 4 of sensor 2, which corresponds to the sum of the complete response of the same sensor and reaches an F-statistic close to 50 (*p* < 0.01).

Each value of the F-statistic has an associated level of statistical significance, i.e., a *p*-value. Small *p*-values confirm that the feature has a high discriminatory potential. To select the most useful sensors in the VOO vs EVOO classification task, the *p*-value was used. So, the sensors with the highest number of significant features (*p*-value equal to or less than 0.01) were selected to build the classifier. Figure 4 shows that the sensors that obtained the highest number of significant features were 2, 7 and 9, all of them with five features with a *p*-value lower than 0.01.

This selection is in line with several research studies in which sensor S2 seems to be key in the detection of fruity aromas [25,27,28]. Regarding the second selected sensor, S7, it has been proven that its high performance in olive oil quality assessment is probably related to the presence of defects [20,25]. Finally, the third sensor chosen, S9, appears to be important in differentiating according to varietal aromatic characteristics [28] and also in relation to the defect detection [25].

**Figure 5:**
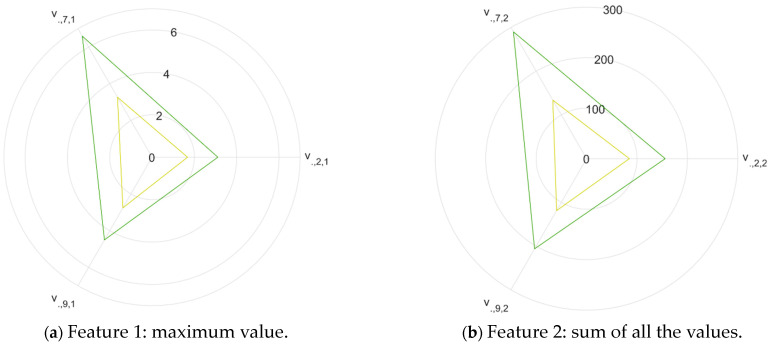

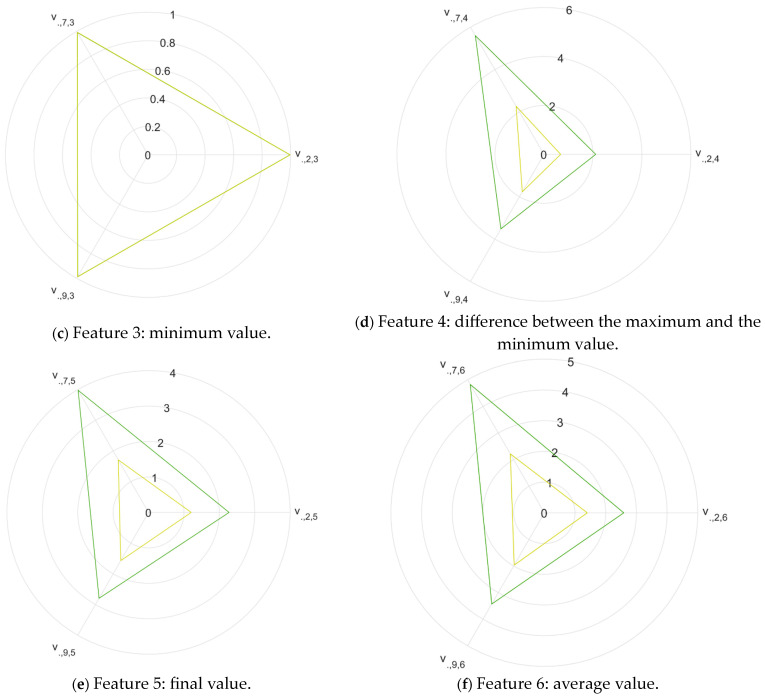
Comparison of the characteristics extracted from the e-nose response for the three most discriminative sensors in the task of classifying between virgin olive oils (yellow line) and extra-virgin olive oils (green line). The numbers indicate the resistance value obtained by each sensor normalized based on the final resistance value of the cleaning phase.

The value of the features extracted from the former sensors is shown in Figure 5. It can be seen that feature number 3 is not useful for separating the two classes of oils. Sensor 7 is also presented as the most discriminative between classes (greater distance between the vertices of the triangles); the better performance for this sensor was also presented in [11], and this same sensor also seemed to have greater activation toward other fruit volatiles such as from berries and mandarins [27,28].

Finally, the five significant characteristics (1, 2, 4, 5 and 6) of each of the three sensors considered most discriminating (S2, S7 and S9) were selected. These 15 features were used to evaluate the performance of a classifier based on a decision tree. It was assessed in terms of true positive (TP) rate. The TP rate is the percentage of observations that have been assigned to an evaluated class when they truly belong to that class. The result, after applying the methodology, was a TP rate of 100%, which means that all the 32 pure samples were correctly classified between EVOO and VOO. This is a good result, as most previous studies had failed to segregate between these classes [8,21]. Furthermore, these results are an improvement on those presented by [20] Martínez Gila et al., 2019, where they attained an up to 85% classification accuracy among the EVOO, VOO and LOO categories.

### 3.2. Detection and Quantification of Olive Oil Dilutions

Once the classifier was fully designed and validated with the pure samples, it was tested on the 160 olive oil dilutions. The purpose of this study was twofold. First, how the e-nose can detect the threshold by which an olive oil is classified as EVOO or VOO was studied. Second, we analysed how the fruity aroma and the types of defects present in the VOOs could interfere in this threshold. Under the hypothesis that an EVOO mixed with a VOO already has a potentially detectable defect, it is considered from now on that a mixture is correctly classified as a VOO according to the classification model executed by the electronic nose.

**Figure 6:**
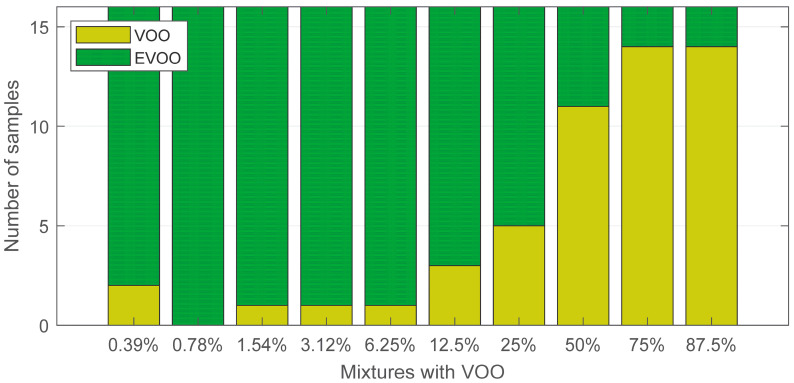
Classification results for all the olive oil dilutions.

For the first study, all the olive oil dilutions were considered, without segregation by fruitiness or defect type. Figure 6 shows the classification results. 

Most of the samples were classified as VOOs for dilutions above 50%. In fact, 14 of the 16 samples were labelled as VOOs for 75% and 87.5% dilutions. In the case of the 50% dilutions, 11 of the 16 samples were labelled as VOOs. As was to be expected, the number of samples labelled as a VOO was lower as the amount of VOO decreases in the dilution. For the 25% and 12.5% dilutions, 5 and 4 of the 16 samples were classified as VOOs, respectively.

Results also indicate that the e-nose can be sensitive to even a small quantity of defects, e.g., in the case of dilutions less than 12.5%. For dilutions of 1.54%, 3.12% and 6.25%, 1 of the 16 samples was identified as a VOO. To conclude with this analysis, none of the samples were identified as VOOs for the 0.78% dilutions, and 2 out of 16 samples were labelled as VOOs for the 0.39% dilutions. The latter case may be due to the threshold tolerance of the classification model.

**Figure 7:**
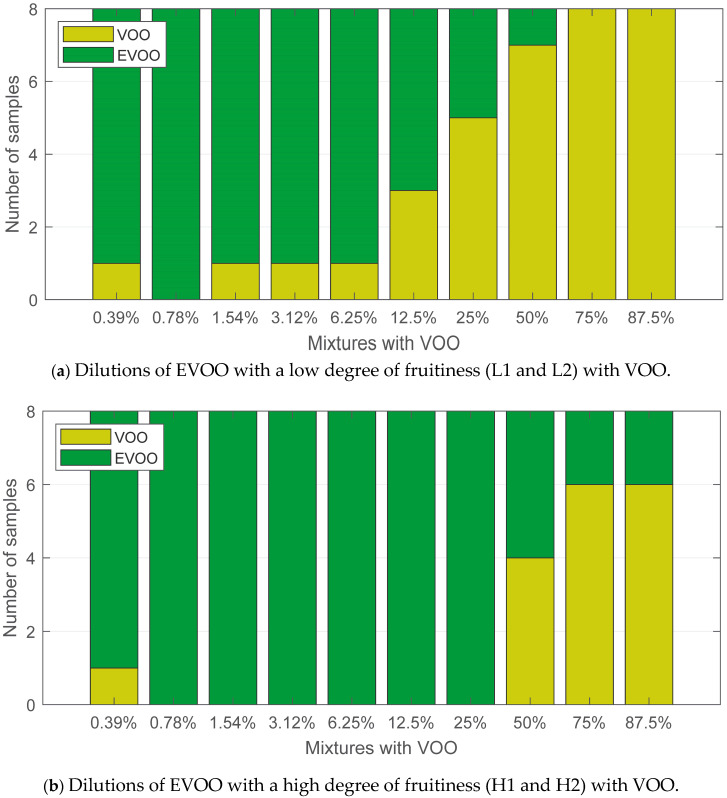
Classification results for olive oil dilutions segregated by the degree of fruitiness.

The classification results shown in Figure 7 highlight the clear influence of the degree of fruitiness on the quality assessment of olive oils. Dilutions prepared with L1 and L2 EVOO samples (Figure 7a) had better results in the classification.

For the 75% and 87.5% dilutions, the eight oils were labelled as VOO, and for the 50% dilutions, seven of the eight samples were correctly classified. As was the case when analysing all the samples, even dilutions with a small amount of VOO were properly labelled.

This happened in percentages below 12.5%, where one of the eight dilutions was labelled as VOO. The detection threshold worsened for dilutions with highly fruity EVOOs. Six of the eight samples were correctly classified for the 75% and 87.5% dilutions. All the dilutions below 50% were classified as EVOO except for one sample from the 0.39% dilution (Figure 7b). Similar to the results shown in the previous figure, this issue may be due to the threshold tolerance of the classification model.

**Figure 8:**
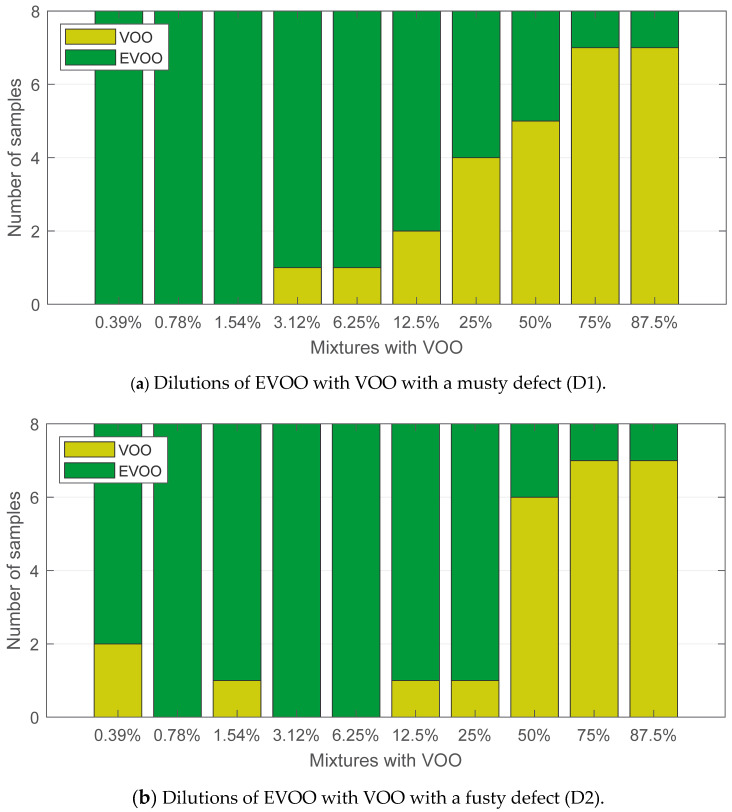
Classification results for olive oil dilutions segregated by the type of defect.

The influence of the defect type in the detection threshold can be analysed on the basis of the results shown in Figure 8. The e-nose is more reliable in detecting the musty defect, as it exhibits a linear response (Figure 8a). The detection started with the 3.12% dilutions, and for percentages above a 25% dilution, more than half the samples were labelled as VOO. From the analysis of Figure 8b, it can be inferred that a VOO with a fusty defect is slightly easier to mask in a mixture. Indeed, eight samples with VOO (musty defect) concentrations lower than 50% were detected as VOO, while in the case of VOO with a fusty defect, the number was five.

**Figure 9:**
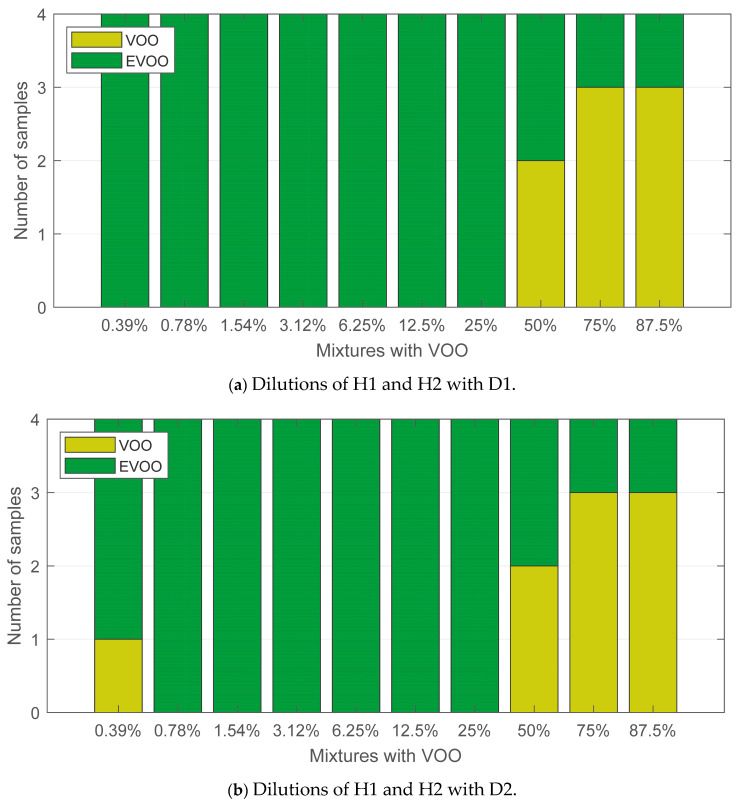

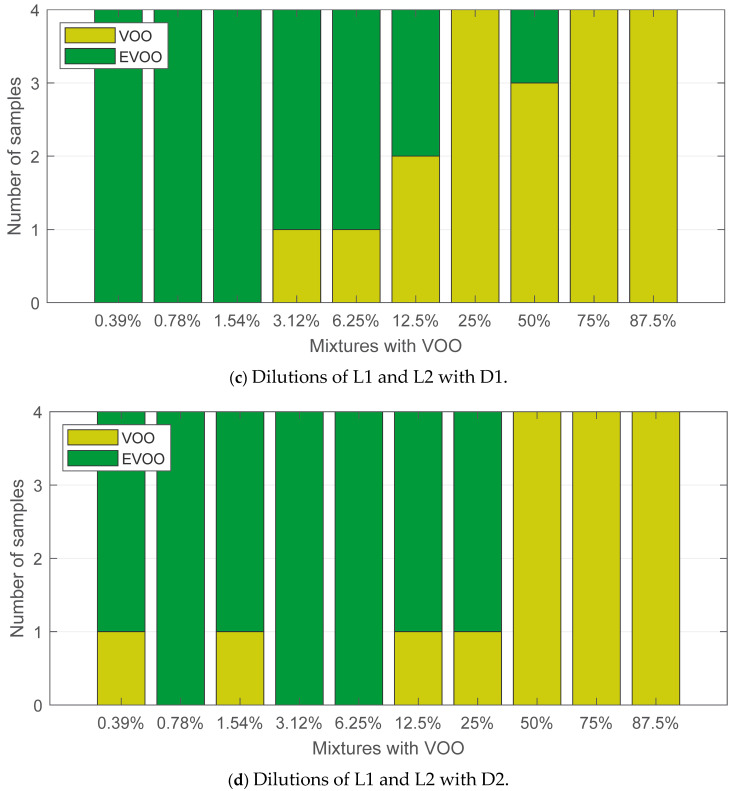
Classification results for olive oil dilutions segregated by the degree of fruitiness and the type of defect.

Finally, the interactions between the degree of fruitiness in EVOO and the types of defects in VOO are shown in Figure 9. As already stated in the discussion of Figure 7, the degree of fruitiness had a great influence on the olive oil quality assessment. Dilutions of EVOOs with a high level of fruitiness (Figure 9a,b) showed a similar response, regardless of the type of defect in the VOO. The opposite is true in the case of dilutions with EVOOs with a low degree of fruitiness. Dilutions with VOO with a musty defect (Figure 9c) were sensed in a higher number of samples than those ones with a fusty defect (Figure 9d). In particular, it was detected from 3.12% dilutions. As shown in Figure 8b, the e-nose displayed random behaviour for dilutions below 12.5%. The same effect can be seen in Figure 9d.

It is worth noting that the intensity of the defects in the VOOs utilized for the dilutions was 2.5 or below. Other research studies devoted to detecting defects in virgin olive oils with the e-nose technology have analysed dilutions produced with VOOs with a much higher intensity of defects. These were always above 3.5, and in some of cases, were 6.9 or even 9 [16,22,23]. In light of the results presented, it can be safely concluded that our methodology is an improvement on these previous works as the success rate in the pure samples’ classification, using VOOs with a low defect intensity, was 100%.

Another important finding from this study is the analysis of how the degree of fruitiness of EVOOs affects the attainment of the detection threshold. In an earlier study [16], it was suggested that a defective oil could be added in a 10% proportion to an EVOO to increase the company profits without risking defect detection by an official panel test. This study has demonstrated that this percentage could be higher or even lower depending on the degree of fruitiness of the EVOO and the type of defect of the VOO utilized for the mixture.

## 4. Conclusions

The results of this study have demonstrated the feasibility of using a commercial electronic nose model for the rapid screening of the quality of olive oil produced. By performing this assessment at-line or on-line, oil mills could store the produced oil in tanks with other oils of similar quality. This practice increases the company’s profitability, as the potential quality of the oil is not reduced, and it can be sold at a better price. Knowing the threshold at which an oil will be classified as EVOO or VOO is essential when mixing.

Also, this has been the first study to identify which sensors of a general-purpose e-nose are more discriminative for olive oil quality assessment. With this information, a classification model was designed with a success rate of 100%. The olive oil samples were characterised by different levels of fruitiness, in the case of EVOOs, and by the different types of defects, at a very low intensity, that affected the VOOs. The identification of the most discriminative sensors for olive oil quality (S2, S7 and S9) could also be very interesting for the construction of low-cost specific-purpose electronic noses. This valuable information could reduce the overall cost of the device, as it is directly related to the number of sensors included in it.

Finally, this work has analysed how the level of fruitiness of EVOOs and certain types of defects present in the VOOs influence the threshold above which the quality of the olive oil is set. In order to analyse the influences of fruitiness and defects on the detection threshold, a set of dilutions between pure oil samples was created. From the analysis of the dilutions, it can be concluded that the quantity of VOO in the mixture can be higher for those samples prepared with EVOOs with a high level of fruitiness. It was possible to add up to 25% of the VOO to the mixture without it being identified by the sensor. For the two types of defects affecting VOOs, the musty defect was the easier to detect. The e-nose had a linear response for this type of defect. In addition, samples of dilutions with only 3.12% of VOO in a EVOO with a low degree of fruitiness were labelled as VOO.

Although the results acquired are positive, the proposed methodology is dependent on the experimental set-up used. To transfer the knowledge acquired in a future industrial facility, it will be interesting to investigate deep neural network models combined with transfer learning methods.

## Figures and Tables

**Table 1:** Sensory characterisation followed by the method described (IOC, 2018).

Quality	Code	Fruitiness Level	Defect Level	Type of Defect
EVOO	H1	5.7	-	-
EVOO	H2	5.6	-	-
EVOO	L1	3.3	-	-
EVOO	L2	3.2	-	-
VOO	D1	2.4	2.5	Musty–Mouldy
VOO	D2	2.4	2.4	Fusty–Muddy

**Table 2:** Combinations between the EVOOs and VOOs for the set of mixtures.

		EVOO			
		H1	H2	L1	L2
VOO	D1	H1D1	H2D1	L1D1	L2D1
	D2	H1D2	H2D2	L1D2	L2D2

**Table 3:** Details of the dilution series to create the different samples.

Identifier	Proportion	Result	
		VOO	EVOO
S1	2.5 g EVOO + 2.5 g VOO	50%	50%
S2	2.5 g S1 + 2.5 g EVOO	25%	75%
S3	2.5 g S2 + 2.5 g EVOO	12.5%	87.5%
S4	2.5 g S3 + 2.5 g EVOO	6.25%	93.75%
S5	2.5 g S4 + 2.5 g EVOO	3.12%	96.88%
S6	2.5 g S5 + 2.5 g EVOO	1.56%	98.44%
S7	2.5 g S6 + 2.5 g EVOO	0.78%	99.22%
S8	2.5 g S7 + 2.5 g EVOO	0.39%	99.61%
S9	2.5 g S1 + 2.5 g VOO	75%	25%
S10	2.5 g S9 + 2.5 g VOO	87.5%	12.5%
S11	5 g EVOO	0%	100%
S12	5 g VOO	100%	0%

## Data Availability

Data are contained within the article.
